# ProPepper: a curated database for identification and analysis of peptide and immune-responsive epitope composition of cereal grain protein families

**DOI:** 10.1093/database/bav100

**Published:** 2015-10-08

**Authors:** Angéla Juhász, Réka Haraszi, Csaba Maulis

**Affiliations:** ^1^Applied Genomics Department, MTA Centre for Agricultural Research, Brunszvik u. 2, Martonvásár, 2462, Hungary,; ^2^Campden BRI, Station road, Chipping Campden GL55 6LD, UK and; ^3^Hegybiro 4, 1221, Budapest, Hungary

## Abstract

ProPepper is a database that contains prolamin proteins identified from true grasses (*Poaceae*), their peptides obtained with single- and multi-enzyme *in silico* digestions as well as linear T- and B-cell-specific epitopes that are responsible for wheat-related food disorders. The integrated database and analysis platform contains datasets that are collected from multiple public databases (UniprotKB, IEDB, NCBI GenBank), manually curated and annotated, and interpreted in three main data tables: Protein-, Peptide- and Epitope list views that are cross-connected by unique identifications. Altogether 21 genera and 80 different species are represented. Currently, the database contains 2146 unique and complete protein sequences related to 2618 GenBank entries and 35 657 unique peptide sequences that are a result of 575 110 unique digestion events obtained by *in silico* digestion methods involving six proteolytic enzymes and their combinations. The interface allows advanced global and parametric search functions along with a download option, with direct connections to the relevant public databases.

**Database URL:** https://propepper.net

## Introduction

Cereals serve as one of the most important energy sources in our daily nutrition all over the world. One of the most produced cereals, wheat grains, are consumed in different forms including leavened and flat bread, pastry, noodles or pasta. The quality requirements of these products strongly depend on the amount and composition of prolamins, the storage proteins of the wheat seed. Their unique characteristics, the high proline and glutamine content stored in the form of short repetitive sections in their protein sequence and the significant number of cysteine residues ensures the compact storage of nutrients that is then utilized during the seed germination. Moreover, these proteins are responsible for the unique structure of bread or pasta dough. The resulting protein network, also called gluten, is composed from unique prolamin proteins and is stabilized by the intra- and intermolecular disulphide bonds formed between the cysteine residues. Prolamins are responsible for severe health problems, such as celiac disease (CD) and partially for wheat allergies (WA). Prolamin proteins, such as high and low molecular weight (HMW and LMW, respectively) glutenins, alpha-, gamma- and omega gliadins share high degree of sequence similarity thus making the precise identification of unique alleles challenging. The detection and especially the quantification of gluten proteins are extremely important not only due to their direct effect on end-use quality but also for food safety reasons. Grain composition varies between cereal genotypes and therefore leads to methodological problems in food allergen research and genotype selection in breeding for quality. The high sequence similarity and multi-species origin of prolamins coupled with limitations in the available methodologies (1) make the exact identification of proteins that trigger health problems and their genotypic frequency, variability and stability difficult to determine. High-resolution methods such as mass spectrometry (MS) require accurate molecular quantitative relationships between prolamin peptide biomarkers and the final gluten/prolamin content to relate the detection of peptide mass to their protein sources. These quantitative relationships however are difficult to establish due to genotypic and environmental variability. In addition, the significantly higher portion of proline and glutamine residues has led to poor digestibility by trypsin, one of the most commonly used enzyme in MS-based proteomics. Other enzymes (e.g. chymotrypsin, thermolysin) and enzyme combinations (e.g. LysC+trypsin) were found to work better to obtain prolamin peptides (2–4) and thus introduced further challenges such as the optimization of enzymatic digestion prior to liquid chromatography mass spectrometry (LC-MS) analyses or processing of mass spectra with bioinformatics softwares that are usually optimized for the use of trypsin. To assist peptide biomarker search, epitope mapping, protein selection and medical studies, a database (ProPepper, https://propepper.net) was developed to contain members of the prolamin superfamily proteins identified from *Poaceae* species, peptides obtained with single and multi-enzyme *in silico* digestion as well as linear epitopes responsible for wheat-related food disorders. This article introduces the content of this database and its potential use and highlights some areas of application. The protein, peptide and epitope sequences are manually curated and annotated from well-recognized databases (e.g. UniProt, IEDB, NCBI GenBank) and scientific publications by the expert authors.

## Methods and materials used for database development

### Data collection, data categories and curation

The ProPepper is a metadatabase that contains three main datasets (proteins, peptides and epitopes). Complete protein sequences, all members of the prolamin superfamily isolated from different *Poaceae* species were retrieved from the UniProt database. Sequences were aligned for a precise identification of the protein types (e.g. alpha-, gamma- and omega gliadins, x- or y-type HMW-glutenins, i-, m- or s-type LMW glutenins, etc.). Misannotations were manually corrected and information related to chromosomal location, origin and allele were automatically fetched from UniProt, NCBI GenBank information and from published results. Chromosomal location of proteins originated from *Triticum aestivum* and *T.* *turgidum* was determined using BLAST algorithm against the published genome sequence data (www.wheatgenome.org and plants.ensembl.org). Alleles of HMW glutenin subunits (*Glu-1* loci), LMW glutenin subunits (*Glu-3* loci) and alpha-, gamma- and omega gliadins encoded at the *Gli-1* and *Gli-2* loci were annotated for *T.* *aestivum* genotypes using the gluten allele databases of Békés and Wrigley (5) and Metakovsky *et al.* (6). BLAST analysis was also used for the identification of *Glu-1*, *Glu-3*, *Gli-1* and *Gli-2* allelic composition of hexaploid wheat genotypes. Epitope information is retrieved from the Immune Epitope Database (IEDB, www.iedb.org) and published CD-specific core epitope collections (7, 8). Epitopes collected from IEDB were filtered using *Homo sapiens* as host organism and *Poaceae* as allergen source. Cereal pollen allergens were excluded from the analysis. Proteins with 100% identity to a protein with known allelic data were annotated as the hit sequence. Proteins present in multiple alleles (like some of the proteins in the LMW glutenin group) were assigned to multiple alleles.

In the frame of the development of the ProPepper database, an independent application tool [Protein Digestion Multi Query (PDMQ)] was developed for *in silico* digestion of the protein dataset (9). The tool applies cleavage rules as published on the ExPasy Peptide cutter web application tool (10) with the advantage of using multiple enzymes simultaneously. Different sets of enzymes and enzyme combinations, all potentially relevant in gastrointestinal digestion or in MS-based protein analyses are used in the *in silico* digestions. Trypsin (TR), chymotrypsin-low specificity (CTR), pepsin-pH 1.3 (PEP), thermolysin (TLN), LysC and proteinase K (PROK) were used on their own in single-step digestions and also in several combinations. Subsequent multi-enzyme digestions (enzymes applied in consecutive steps) and simultaneous multi-enzyme digestions (enzymes applied together in the same step) were applied using the PDMQ application as shown in Table 1. Digestion events related to a peptide entry obtained with these *in silico* digestion methods are marked in ProPepper as level 0 or 1 meaning that the peptide is a direct result of a protein digestion or was obtained from another peptide as a result of digestion step 2, respectively.

**Table 1.** *In silico* enzymatic digestions as applied to all protein entries in ProPepper resulting the peptide sub-database

Enzyme combination	Digestion step 1	Digestion step 2
CTR	CTR	
CTR-PEP	CTR	PEP
CTR-TR	CTR	TR
LysC	LysC	
LysC+TR	LysC+TR	
LysC+TR+CTR	LysC+TR+CTR	
PEP	PEP	
PEP-CTR	PEP	CTR
PEP-CTR+TR	PEP	CTR+TR
PEP-TR	PEP	TR
PROK	PROK	
TLN	TLN	
TR	TR	
TR-CTR	TR	CTR
TR-PEP	TR	PEP

TR, CTR, PEP, TLN, LysC and PROK were used in single-step and multi-step enzymatic cleavage.

The ProPepper database is a continuously curated database. New protein sequences are collected from the UniProt database four times a year. Annotation information of unique protein sequences is fetched regularly from the NCBI GenBank completed with annotations gained from BLAST analyses or the Gluten allele database (5). New epitope and immune response data from the IEDB database are also updated with the same frequency as the new sequences and annotations. The authors provide technical and scientific support, related to the datasets and the use of the database, continuously.

### Implementation and structure of the database

The data in ProPepper are stored in MySQL relational database system; the software logic was implemented in PHP. The Web interface was developed using PHP and JavaScript. AJAX was used to asynchronous data sending and retrieval. Current versions of all major browsers are supported.

**Figure 1.**
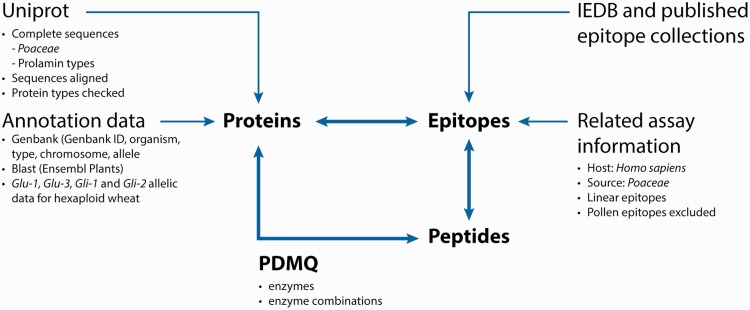
Database composition and analysis pipeline.

The integrated database and analysis platform contains datasets that are collected from multiple public databases and interpreted in three main data tables: Protein-, Peptide- and Epitope list views that are cross connected by unique identifiers (IDs) (Figure 1).

**Figure 2.**
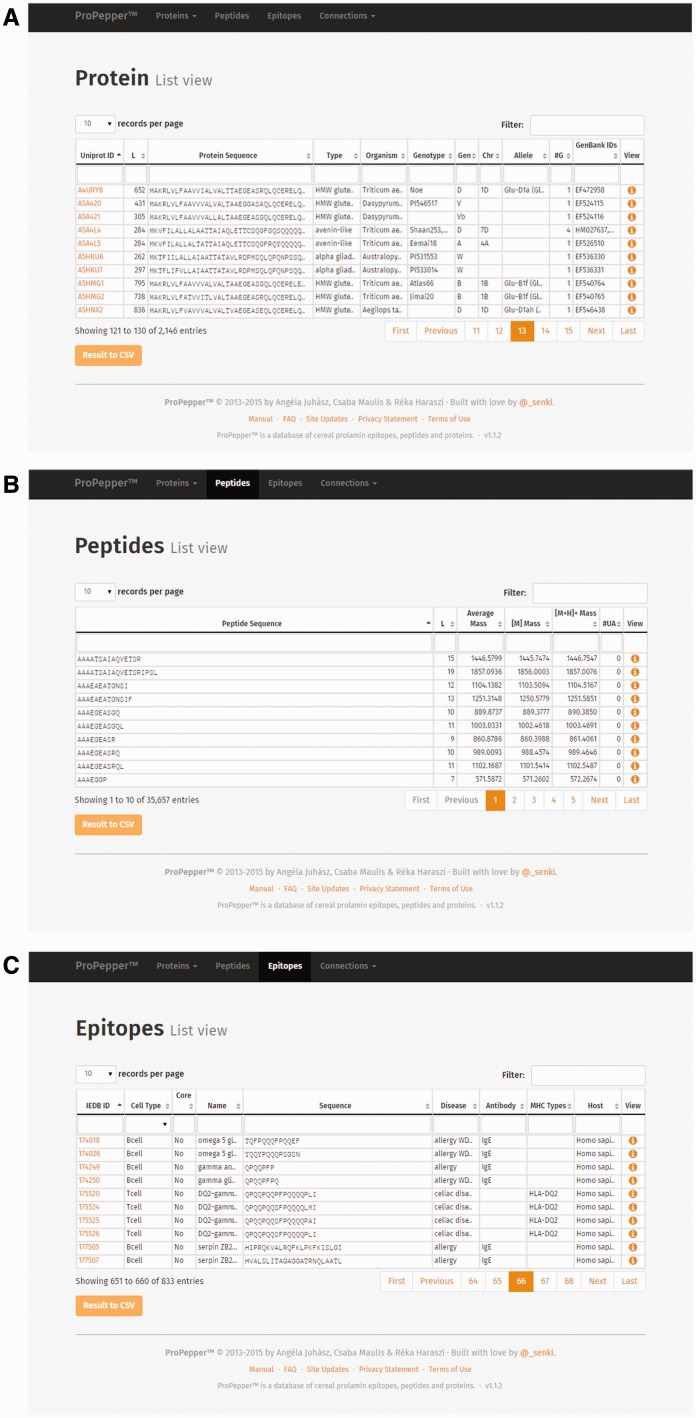
List views as displayed in ProPepper: (**A**) Protein list view, (**B**) Peptide list view and (**C**) Epitope list view.

The Protein list view contains the UniProt ID, which is directly linked to the UniProt database (Figure 2A and B). Information related to a protein entry also includes length (L), protein sequence, protein type, organism(s) and reported genotype(s) containing that protein, as well as further GenBank data (genome, chromosome and allele). GenBank IDs referring to the coding genes are also presented in a separate GenBank annotation table.

The Peptide list view contains the peptide sequence, the peptide length, different mass values (Average Mass [M], Monoisotopic Mass [M+H] and singly-charged monoisotopic mass [M+H]^+^) and the number of unrecognized amino acids (displayed in column #UA in the ProPepper) (labelled as X in the protein sequence) (Figure 2B).

The Epitope list view contains the IEDB epitope ID where available, the cell type directly bound to the epitope (T cell or B cell), information whether the epitope is a core epitope (only for nine amino acid long CD-related epitopes), name and sequence of the epitope, caused disease, the related antibody heavy chain (IgE, IgG, IgA for B-cell epitopes only), MHC serotype and host organism, respectively (Figure 2C).

**Figure 3.**
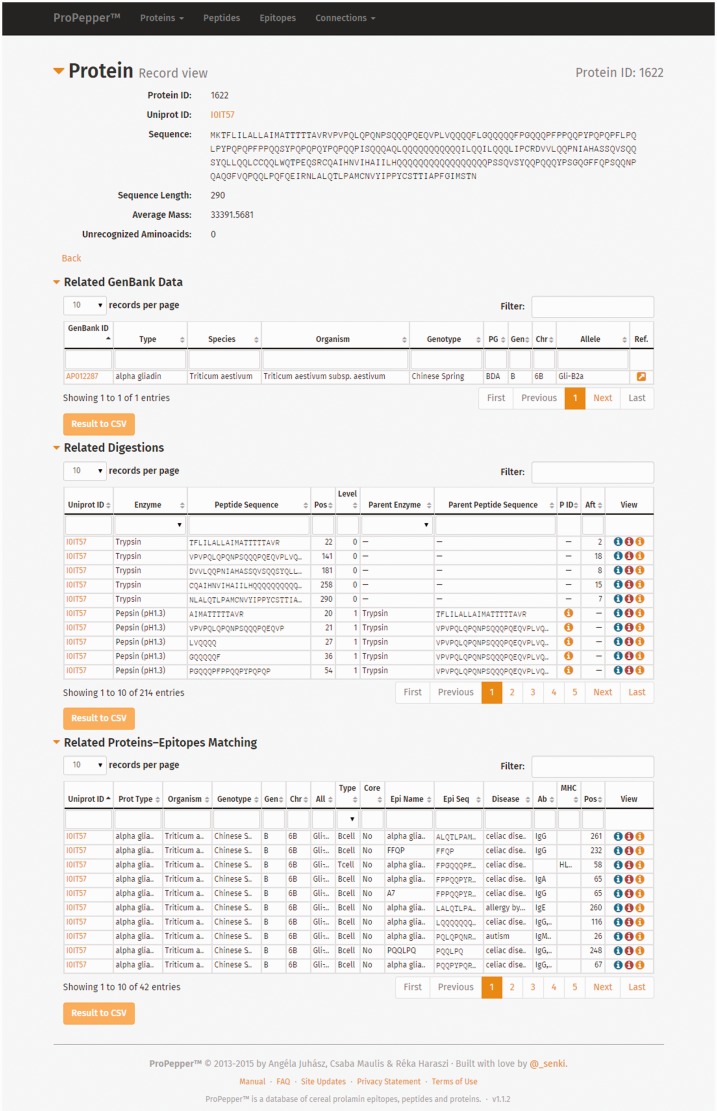
Individual record views as displayed in ProPepper: Protein record view and related tables: GenBank data, Digestions and Proteins-Epitopes matching tables. The Propepper contains Peptide record view and Epitope record view similarly to the Protein record view.

Individual Protein record view contains information of the protein entry, the related GenBank data, the related digestions and the related protein–epitope matching hits (Figure 3). Related digestion tables in the Protein record view include enzymes used for digestion, protein IDs that contain the particular peptide, starting position of the peptide in the protein sequence, level of digestion and enzyme and peptide sequence used in the previous (Parent) digestion. Related peptide–epitope matching table in the record view contains epitope and immunoassay-specific information.

Individual Peptide record view contains information of the peptide entry, the related digestions and the related peptide–epitope matching pairs. Individual epitope record view contains further details related to the source and characteristics of the epitopes, as well as IDs of the epitope and the related immunoassays with a direct link to the IEDB database and their references.

**Figure 4.**
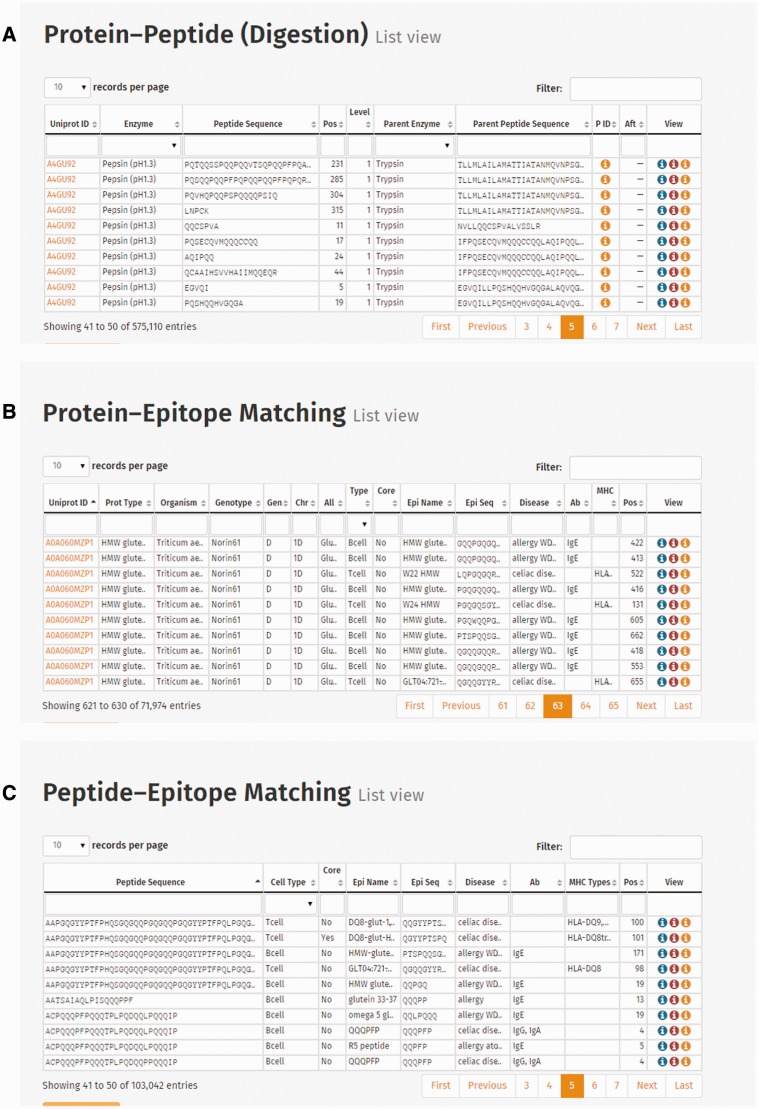
Screenshots of connection tables: (**A**) Protein–Peptide connection (digestion) list view, (**B**) Protein–Epitope matching list view and (**C**) Peptide–Epitope matching list view.

Connection list views represent digestion events [Protein–Peptide connection (Figure 4A), Protein–Epitope matching (Figure 4B) and Peptide–Epitope matching data (Figure 4C)]. The Protein–Peptide connection table provides information about the UniProt ID, enzyme, peptide sequence, position of the sequence, level of digestion, parent enzyme and parent peptide sequence and IDs of preceding and following digestion events presented. Protein–Epitope matching table presents UniProt ID, protein type, origin information (organism, genotype, genome, chromosome and allele) and information of the epitope (cell type, core epitope, epitope name, sequence, the caused disease, antibody, MHC serotype and epitope position). Peptide–Epitope matching table represents the epitopes resistant to digestion, and their harbouring peptides, including peptide and epitope sequence information, cell type reactive to the epitope, disease caused by the epitope, immunoglobulin antibody or MHC serotype and position of the epitope in the peptide sequence.

### Use of ProPepper resource

The database currently (using the UniProt datasets available at January 2015) contains data from three tribes of true grasses (*Poaceae*), namely *Triticeae*, *Avenae* and *Brachypodieae*, from which the number of genera of *Triticeae* is the most abundant. Altogether 21 genera and 80 different species are represented, from which 19 genera are member of the *Triticeae*. *Triticum* species take more than the 52% of the entire dataset. Cereal species such as *Aegilops tauschii*, *T.* *turgidum*, *T.* *urartu* and *T.* *monococcum* are also represented with a significantly high count of sequences. The analysed protein families include HMW-glutenins, LMW-glutenins, alpha-, gamma-, delta- and omega-gliadins, B-, C- and D-hordeins, gamma- and omega-secalins, avenins, avenin-like proteins and farinins. Subtypes of HMW glutenins (x- and y-types) and LMW glutenins (i-, m- and s-type) are also distinguished in the ProPepper database.

Currently, the database contains 2146 unique and complete protein sequences and 35 657 unique peptide sequences. The number of unique peptides in ProPepper is a result of 575 110 unique digestion events. The complexity of the peptide database is reflected in the diversity of peptides in the three most relevant genera containing various numbers of *Triticum, Hordeum* and *Secale* species across protein types and as cleaved by various enzymes. Comparing these three species in Table 2, it is evident that enzymes are specific in obtaining peptides from certain protein types and species.

**Table 2.** Number of peptides from (A) *Secale*, (B) *Hordeum* and (C) *Triticum* species that are cleavable with various enzymes and belong to a group of protein type

Protein type	CTR	CTR+TR	LysC	LysC+TR	LysC, TR, CTR	PEP	PROK	TLN	TR	Grand Total
(A)										
Alpha gliadin	915	82		61	7	665	267	255	402	2654
Alpha prolamin	74	8		2	1	63	26	23	28	225
Gamma secalin	1726	162	4	90	21	1210	888	497	545	5143
HMW glutenin x-type	1183	65	12	68	6	1202	486	280	464	3766
HMW glutenin y-type	1278	81	9	89	8	932	425	344	668	3834
Omega secalin	65	19		3	1	73	42	24	12	239
Secalin	2069	252		104	27	1629	1148	622	680	6531
Grand total	7310	669	25	417	71	5774	3282	2045	2799	22 392
(B)										
Avenin-like	84	7	1	3	1	63	26	29	38	252
B-hordein	708	56	1	36	5	612	310	264	339	2331
C-hordein	60	13		1		62	36	16	6	194
D-hordein	536	54	4	27	2	568	215	170	236	1812
Gamma hordein	156	14		18	2	128	58	50	72	498
Hordein	3687	357	19	223	15	3249	1518	1285	1380	11 733
Grand total	5231	501	25	308	25	4682	2163	1814	2071	16 820
**(C)**										
Alpha gliadin	22 893	2473	20	626	3	16 699	7644	6779	7713	64 850
Avenin	3165	305	31	106	41	2395	937	1034	1496	9510
Avenin-like	1250	109	12	35	14	1078	349	378	549	3774
Gamma gliadin	17 687	1626	172	843	207	14 811	6560	4988	6046	52 940
Gamma secalin	70	7		4	1	40	18	17	25	182
HMW glutenin x-type	11 150	769	7	505	25	10 979	4314	3050	5100	35 899
HMW glutenin y-type	7450	582	12	592	48	6218	2681	2179	4279	24 041
LMW glutenin	65	6		3	1	52	23	21	20	191
LMW glutenin i-type	13 968	1080		384	121	6323	4178	4300	3101	33 455
LMW glutenin m-type	42 362	4499	172	1781	448	31 925	13 994	14 487	13 713	123 381
LMW glutenin s-type	8586	959	37	226	76	5819	2779	2905	2104	23 491
Omega gliadin	512	94	2	33	10	745	397	150	176	2119
Secalin	1759	394	3	114	34	1681	980	502	544	6011
Grand Total	131 699	12 968	471	5295	1035	99 432	45 126	41 018	45 233	382 277

TR, CTR, PEP, TLN, LysC, PROK and all relevant enzyme combinations were used for grouping the number of hits.

**Figure 5.**
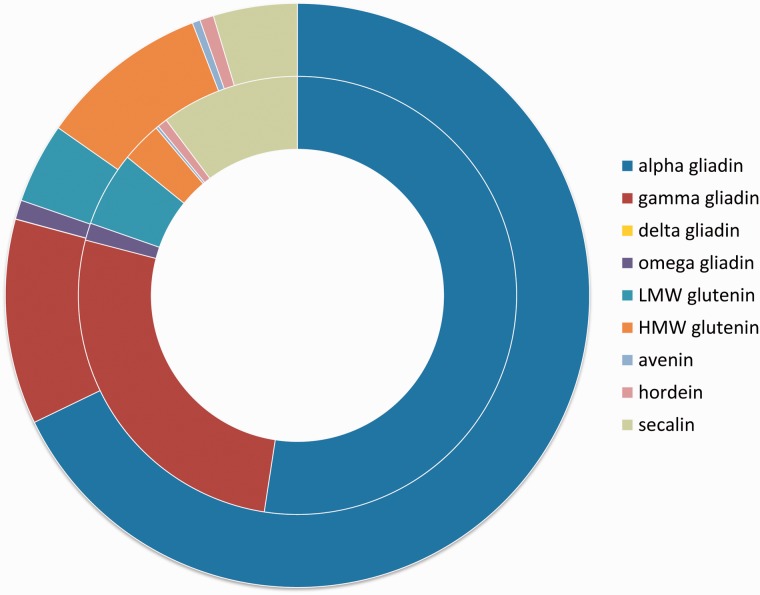
Number of epitopes in the different prolamin protein types. Inner circle shows the distribution of T-cell-specific linear epitope counts in prolamin types represented in the ProPepper database. Outer circle represents the distribution of B-cell-specific linear epitope hits found in the different prolamin types. Prolamin types are labelled by different colours.

The epitope dataset of the ProPepper database contains linear epitopes with proven T-cell- or B-cell-specific immune-activity. Altogether 833 unique linear IEDB epitope records are presented in 1262 immunoassays. From the 833 unique epitopes, 327 belong to gluten-related T-cell epitopes including 35 core epitopes. In total, 499 epitopes are gluten-related B-cell epitopes (Table 3). B-cell epitopes related to allergic responses of wheat, such as allergic asthma or wheat-dependent exercise-induced anaphylaxis (WDEIA), are also differentiated. Some *Poaceae*-specific linear epitopes related to psoriasis, autism, diabetes mellitus or rice allergy are also presented in the database. Number of epitopes can also be summarized in the different protein types of the analysed *Poaceae* genera. A summary of epitope distributions per prolamin type is presented separately for T-cell- and B-cell-specific epitopes in Figure 5.

**Table 3.** Number of B- and T-cell epitopes related to cereal-related food disorders originating from *Poaceae* species

Related disease	B-cell epitopes	T-cell epitopes
Allergy	336		
Allergy	89		
Allergic asthma	56		
Allergy atopic dermatitis	1		
Allergy baker's asthma	6		
Allergy by trigger	59		
Allergy WDEIA	125		
Rice allergy	3	
Celiac disease	161	328
Dermatitis herpetiformis	1	1
Diabetes mellitus	1	1
Autism	2	
Food hypersensitivity	5	
Psoriasis	1	1

Related diseases are labelled as presented in the IEDB database.

Querying the database can be performed at different levels. Besides the main filter, column-based filters are included in all three datasets, and in all tables in list- and record views. Results can be obtained using a rapid search by keywords that represent, e.g. a part of the sequence of a protein, peptide or epitope, a name of an organism or genotype or a chromosome ID. Only hits that contain the typed keyword are displayed in real time. It is possible to filter the results and use a suggested step-by-step approach. For example, searching for ‘A genome’-specific HMW glutenins can be performed by first searching for ‘HMW glutenins’ followed by searching for ‘A’ in the Genome column filter. The results obtained after each search step can be downloaded in csv format and used for further analysis when required. Targeted queries can be performed in order for instance, to analyse prolamin characteristics at species and genotype level; to identify peptides resistant to gastrointestinal enzymes; to identify peptides or epitopes suitable for MS-based marker analyses and to identify epitopes at unique protein or peptide level.

### MS module

**Figure 6.**
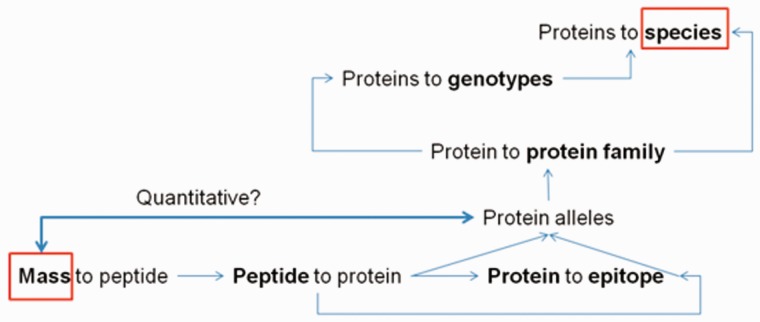
Relationships among peptide, protein and epitope data that can be obtained from the ProPepper database.

The ProPepper database can be a useful tool in the design and evaluation of MS-based proteomics workflow. It is especially challenging when cereal proteins are present in a food as contamination. Particularly important field of such applications is the detection of allergens. The collection of sequence information, the performance of *in silico* digestions, the annotations and BLAST analyses for sequence specificity are all necessary steps in MS-based detection. The ProPepper contains this information for prolamins and that makes it extremely useful to speed up LC-MS applications. The database provides support for the design of a digestion method, the data processing of mass spectra and the peptide matching process of the identified masses. In a MS discovery workflow, the list of identified masses from a mass spectrum needs to be related to a peptide sequence and a protein source. This information is usually in a database that is selected and fed by the user to the search engine of the data processing software when performing LC-MS analysis. The database size and the specificity of the data entries can influence the results of the likelihood-based matching process and the final scores for the protein and peptide hits. This type of measure is usually optimized for peptides obtained from a trypsin digestion, so in case of the application of other enzyme(s) and especially of multi-enzyme digestion, the meaning of this score is limited. The cross connections among peptides, proteins and the annotated data in ProPepper offer the opportunity to relate peptide masses to a cereal species or genotype via the identification of individual peptides and its protein source even at allelic level (Figure 6).

**Figure 7.**
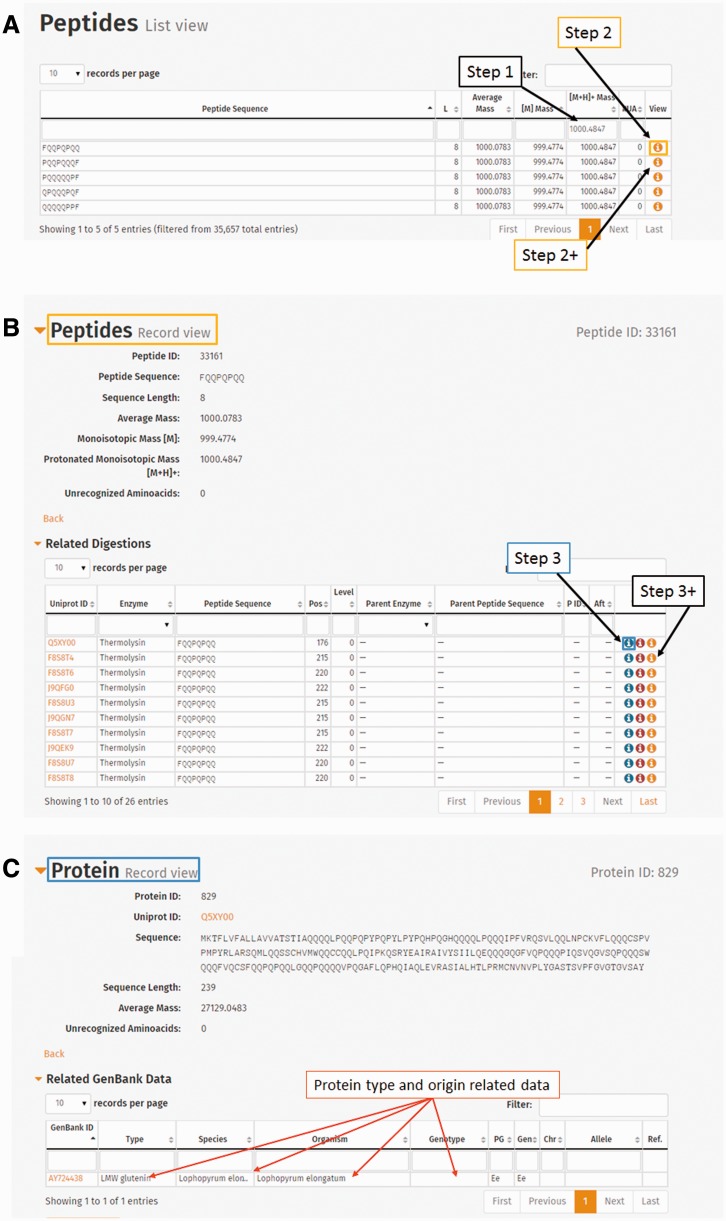
The use of peptide mass entry in the ProPepper database to establish its relevance to peptides, proteins, genotypes and species. (**A**) Entering protonated monoisotopic mass value in Peptide list view. (**B**) Detailed information of a peptide selected from the Peptide list view. Related tables such as ‘Related digestions’ or ‘Related Peptide–Epitope matching’ are also available from this view. (**C**) Detailed information of a Protein by clicking the first icon in the last column (View) of a related digestion entry from (B). The related GenBank data table will give the information of the protein type, organism and genotype (marked with arrows) that contain the particular peptide under investigation.

ProPepper can be a good confirmation tool to double check the specificity of already identified prolamin peptide sequences. Entering the detected mass (e.g. 1000.4847) in the column search box of the singly charged monoisotopic mass [M+H]^+^ in Peptide list view of the ProPepper will reveal all related connections to potential peptide sequences, digestion events, proteins and genotypes. By further selecting a peptide from the hit list, the relevant sequence and other annotated information will be available. Figure 7 shows the steps of such a mass search from this database, a summary of results can be generated as shown in the example in Table 4.

**Table 4.** Summary of the number of proteins and peptides that cleaved by enzyme(s) from the protein. The data also show the number of species, types and genotypes that contain the peptide sequence that is related to the search example of protonated monoisotopic mass 1000.4847

Peptide sequence	Enzyme	Number of proteins	Number of peptides in a protein	Number of species	Number of types	Number of genotypes
FQQPQPQQ	Thermolysin	26	1	3	2	6
PQQPQQQF	Proteinase K	49	1	12	2	10
PQQPQQQF	Pepsin (pH1.3)	3	3	2	2	na
QPQQQPQF	Pepsin (pH1.3)	2	**1**	*Hordeum vulgare*	Hordein	2
QPQQQPQF	Proteinase K	7	1	6	2	2
QPQQQPQF	Chymotrypsin-low specificity, Trypsin	2	**1**	*Hordeum vulgare*	Hordein	2
QPQQQPQF	Chymotrypsin-low specificity	2	**1**	*Hordeum vulgare*	Hordein	2
QQQQQPPF	Chymotrypsin-low specificity	3	4	2	1	2
QQQQQPPF	Proteinase K	3	1	2	1	2
PQQQQQPF	Proteinase K	14	1	3	1	na

An analysis as such can answer, e.g. the following questions:

1. What peptides belong to a detected mass (e.g. 1000.4847) using an enzyme or multiple enzymes?
2. What enzymes can be used to get a particular peptide?
3. How specific is the detected mass for a *Poaceae* species? or
4. How specific is a peptide for a protein type?

The protonated monoisotopic mass 1000.4847 as detected in this example can be present in five prolamin peptide sequences and is obtainable with the range of enzymes as shown in Table 4. The number of hits varies and is a good indicator of the specificity of the peptide for a protein type or species.

The 575 110 unique (non-redundant) digestion events in the ProPepper database include redundant protein–peptide connections that are due to the presence of some protein sequences in multiple genotypes and the multiple prevalence of the peptide within a protein. When the aim is to obtain a specific peptide in a sample for detection or quantification with MS, the digestion process needs to include an enzyme which cleaves out this peptide either directly from a protein sequence or in a subsequent digestion step in a multi-enzyme workflow. The application of proteases in prolamin digestion often followed the route of using trypsin according to the conventional proteomics workflow. Only recently has it been realized that prolamins represent an exception and other enzymes than trypsin may prove to be more efficient. In the current example, when trypsin, chymotrypsin or pepsin is used, QPQQQPQF is present only in barley hordeins in a single copy and in two different proteins. When PROK is used. This peptide is present in six different Poaceae species in seven different proteins representing two types of proteins. Further searches can be done depending on interest towards details of, e.g. what are those two hordeins that contain the QPQQQPQF peptide.

### Epitope module

**Figure 8.**
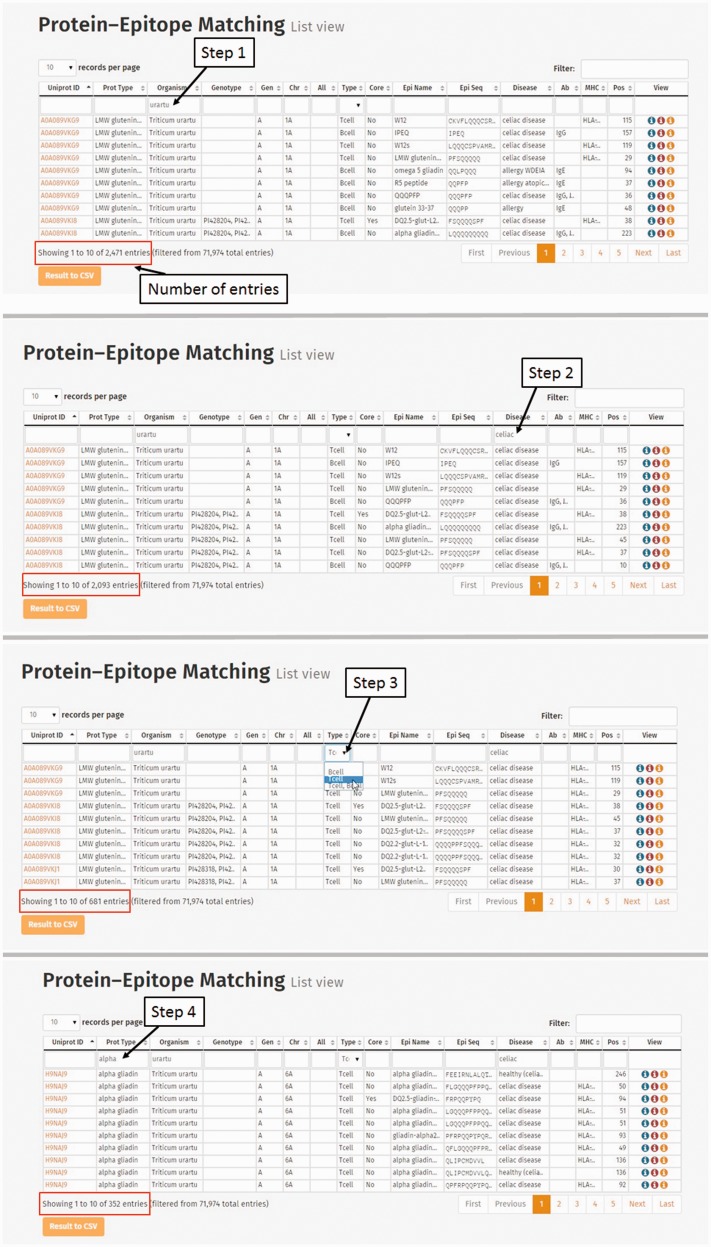
Steps of database query for the analysis of *Triticum urartu* alpha gliadin T-cell-specific epitopes related to celiac disease. Step 1: Selection of *T. urartu* protein sequences from the Protein–Epitope matching list view table. Number of entries representing prolamin protein–epitope matching records is found below the table. (Step 2) *Triticum urartu* protein epitope matches are screened to present only celiac disease-specific hits. (Step 3) Matching records related to T-cell-specific linear epitopes are selected from the Type column. Records representing alpha gliadin-related Protein–Epitope matching hits are narrowed down by entering alpha gliadin into the Prot Type column.

The ProPepper database can be used to evaluate the epitope content and frequency of different cereal species, e.g. the epitope content of A, B and D genome *Triticeae* species. Due to the significant increase in number of patients suffering from different wheat-related food disorders such as CD or WA, the demand to develop wheat genotypes suitable for the special needs of such individuals is constantly increasing. One of the focuses of these developments was to investigate the possibility to use ancient wheats such as einkorn (*T.* *monococcum*) or kamut (*T.* *turgidum* subsp. *turanicum*) as well as wheat genome donor species to produce wheat products with less allergen or toxic epitope content. The main scope of these studies was to characterize the seed storage proteins and their allergen or toxic potential. Some of these studies were focusing mainly on gliadins (alpha and/or gamma gliadins) as these protein families were considered as the primary trigger of CD (8, 11–14). Other studies were investigating the presence of strong allergens such as omega gliadins (15, 16). Prolamin proteins of A, B and D genome species, such as *T.* *aestivum* (ABD), *T.* *turgidum* (AB), and their genome donors *T.* *urartu* (A), *A. speltoides* (S) and *A.* *tauschii* (D) were used in our study to determine whether there is a difference in the epitope count and frequency of epitopes related to CD or WA in prolamin proteins from different species and different genomes. Protein sequences of the following prolamin types were analysed separately: alpha gliadins, gamma gliadins, delta gliadins [a minor prolamin group identified by Anderson *et al.* (17)], LMW glutenin i-type, LMW glutenin m-type, LMW glutenin s-type, HMW glutenin x-type, HMW glutenin y-type, omega gliadin and avenin-like protein. Protein–epitope connection table was used for the analyses. One way to show the difference in the epitope count and frequency of epitopes is to carry out a step-by-step selection process (Figure 8). For example, the species *T.* *urartu* was screened first in the Organism column (2471 protein–epitope matching entries), followed by the search for CD in the Disease column. This resulted in 2093 entries. Among celiac-specific epitope matching hits only those specific for T-cell epitopes (681 hits) were selected and finally alpha gliadins were chosen (352 entries). The result table can be downloaded in csv format for further analysis.

To carry out such a multilevel analysis, the entire Protein–Epitope matching list can be saved in csv format and tools like Pivot tables can be used to summarize entries to reveal complex relationships, e.g. number of CD or wheat allergy-related B- or T-cell-specific epitopes in the different prolamin types at different genome levels.

**Figure 9.**
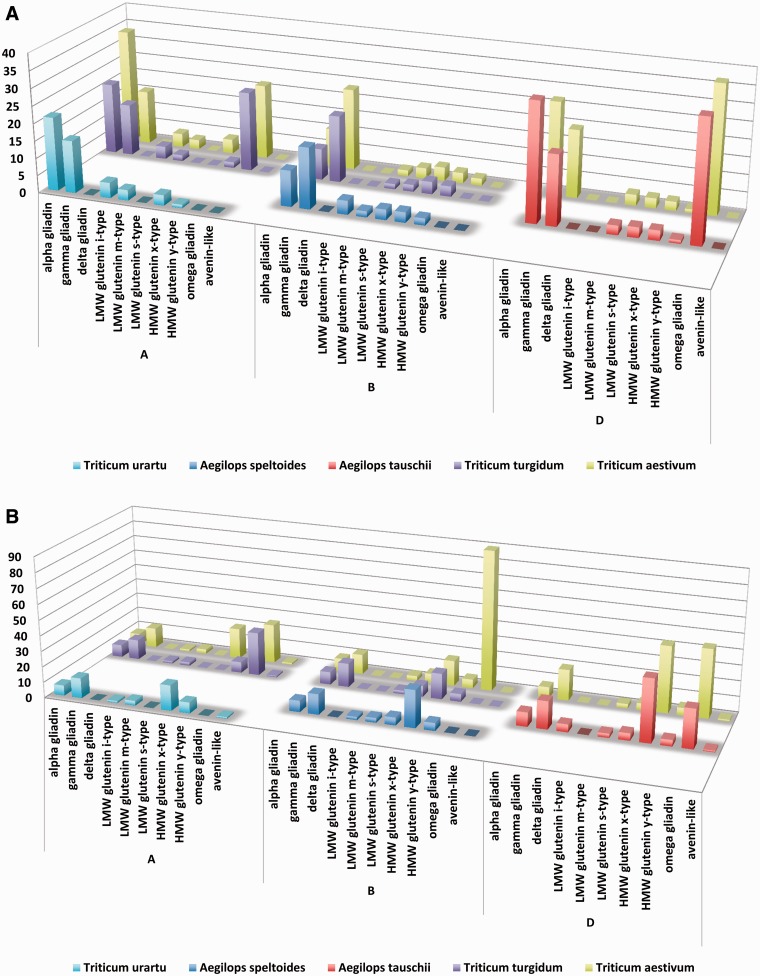
Complex analysis of celiac disease-specific T cell and allergy specific B-cell epitopes in *Triticum aestivum* and their donor species. Epitope counts normalized against the protein number were used to compare epitope density characteristic on different genomes, wheat species and genome donor species. X axes present the analysed prolamin protein types identified in the A, B and D genomes of the different species. Counts of *Aegilops speltoides* (S genome) are presented in the B genome group. Y axes shows the number of epitopes divided by the number of proteins with epitopes as identified from the different prolamin types of the different species. Higher columns represent more epitopes per protein sequence. (**A**) Presence and density of celiac disease-specific linear T-cell epitopes. (**B**) Presence and density of linear B-cell epitopes related to wheat allergies.

Although alpha gliadins were considered for decades to be the primary trigger of gluten toxicity, our results have also confirmed that CD-specific epitopes are common in most of the prolamin protein types (Figure 9A). When the aim is to compare the epitope contents of the prolamin types encoded at the different genomes, one of the possibilities is to normalize the epitope counts to the number of proteins containing the relevant epitope type (i.e. CD-specific T-cell epitopes). Using this normalized dataset, the bias due to the different number of publicly available protein types was eliminated and epitope content of the prolamin types originating from different genomes or species can be compared. Although without the expressional profiles this normalized value is not suitable to directly compare the allergenicity of the proteins, it can serve important information on the prevalence of the different epitopes in the prolamin types. For protein records with allelic information, this analysis can be used to relate epitope counts to allelic differences. Based on this dataset, prolamin types encoded at the D genome contain more T-cell epitopes, followed by the A genome and the B genome (Figure 9A). However, when epitope contents of the different prolamin types are compared for each genome separately in the D genome species (*A.* *tauschii* and *T. aestivum*) omega gliadins and alpha gliadins contain the highest number of epitopes. Among the A genome species alpha gliadins and gamma gliadins contain the most epitopes; however in the polyploid species, omega gliadins are also rich in epitopes. The lack of epitopes in *T.* *urartu* omega gliadin sequences is the result of complete lack of omega gliadins from the public protein databases.

**Figure 10.**
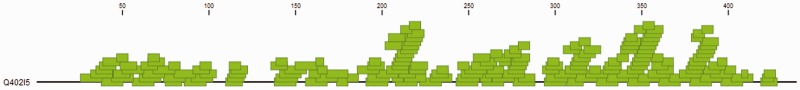
Coverage of wheat allergy related B-cell-specific epitopes in a highly allergen omega-5 gliadin (UniProt ID Q402I5).

When B-cell-specific allergy-related epitope contents are compared, omega gliadins and HMW glutenins contain the most number of epitopes (Figure 9B). Among these sequences an omega gliadin (UniProt ID Q402I5) encoded at the B genome of a *T.* *aestivum* genotype contains 90 epitopes in its sequence. These 90 wheat allergy-related epitopes were downloaded from the ProPepper database and were mapped to the sequence using the Motif search algorithm of the CLC Main Workbench 7.6.1 software package (Qiagen Aarhus A/S) (Figure 10). The strongly overlapping epitopes that cover almost the entire protein sequence are due to the fact that most of them were identified in a systematic study of Battais *et al.* (18) and uploaded to the IEDB database.

When types of prolamin proteins related to the different food disorders are compared, omega gliadins have elevated epitope contents specific both for allergies and CD. However, while in CD sulphur-rich prolamins (alpha gliadins, gamma gliadins and all three sub-types of LMW glutenins) can play a significant role, in WDEIA and other types of WA, these prolamin types may have less importance due to the reduced number of epitopes present in their sequences. In contrast, HMW glutenin subunits contain significantly more allergy-related epitopes (Figure 9B). However, to obtain toxicity and allergenicity values of these proteins, the epitope counts gained from the ProPepper database should be multiplied by the expression values obtained from different proteomic studies. Depending on the individual expression values and the glutenin and gliadin allelic composition of the genotype, the order of significance of protein types can be different.

### Comparison with other available resources focusing on prolamin peptide and epitope analysis

There are multiple web-based allergen databases available that are widely used by scientist interested in allergen identification, analysis and food safety issues. Based on the structure and content of the databases, they can be divided into two main types: allergen databases that provide credible source of known, peer-reviewed allergen proteins and/or epitopes of food materials both of animal and plant origin including information on clinical and physiological aspects of the allergen. Database, such as Allergome (www.allergome.org) and the InformAll Allergenic Food Database (www.inflammation-repair.manchester.ac.uk/informAll/), represents this type of databases. Generally, they cover broad spectra of allergens and provide information on caused disease, symptoms, immunoassays, detection methods, biological function and structure or purification methods of the causative allergen. The second type of allergen databases is rather sequence based and focuses on the molecular features of allergenic proteins, including sequence and structural information of the epitope or the allergenic protein. Post-translational modifications and prediction of allergenicity based on sequence alignments are available from databases such as AllergenOnline (www.allergenonline.org), Immune Epitope Database and Analysis resource (IEDB, www.iedb.org), Allergen Database for Food Safety (ADFS, allergen.nihs.go.jp/ADFS) or Allermatch™ (www.allermatch.org). Some of these databases, such as IEDB, provide different algorithms and learning datasets to predict whether a custom protein shows features of known allergens or not. There are also prediction tools often used to predict the presence or absence of linear or structural epitopes following physico-chemical features of the protein obtained from the amino acid sequence of the protein, sequence identity or the relevant FAO/WHO allergenicity rules based on sequence homology (19). The common characteristics of these databases are that they are summarizing the knowledge of known allergen proteins and epitopes in a broad range of allergen food sources. Most of them also contain information on cereals, including food-related cereal allergens, or respiratory allergens. However, the number of known cereal allergens and epitopes in these databases is limited.

The major advantage of the ProPepper database is that it makes use of some unique features of the prolamin super-family, specifically their high sequence similarity and conserved domain structure and structural similarity. It is known that some of these closely related homologues can share immunological cross-reactivity, such as ability to bind to the MHC II cells or the ability of IgE binding (20, 21). For the best of our knowledge, ProPepper is the first tool that relates the sequence similarity of the different prolamin protein families to the presence or absence of specific immune-reactive epitopes and marker peptides. The screening method applied in ProPepper supports the 100% sequence identities using known epitopes identified from different prolamin sources. Therefore, the presence of an epitope, e.g. from alpha gliadins can also be characteristic on a closely related prolamin type such as gamma gliadins. Both the peptide and the epitope search are based on the 100% sequence alignment when mapping them against the curated gluten protein dataset. The presence of the same peptides or epitopes in two different protein types can represent an evolutionary relationship, whereas unique peptides might represent prolamin type or species-specific protein groups. Therefore, this database can be useful in the development of biomarkers that are specific for certain species, organisms or prolamin types.

## Conclusions and perspectives

ProPepper is a unique sequence similarity-based database that builds upon the common physico-chemical features, the shared biological function and related evolutionary origin of cereal prolamin protein families represented in their similar amino acid composition, high sequence homology and structural similarity. These features are responsible for several difficulties in their analytics and justified the need to develop a regularly maintained, manually curated expert database of prolamin proteins, peptides and epitopes that combines the knowledge of several well-known and acknowledged databases in the fields of protein and allergen research and peptide analysis. It provides a great tool for proteomics, MS and clinical experts that are dealing with prolamins, this unique and complex protein family.

At the moment only the main prolamin protein families, namely alpha-, gamma and omega gliadins, HMW and LMW glutenins are included. Further protein families, also members of the prolamin superfamily, such as puroindolines, nsLTPs (non-specific lipid transfer proteins) and alpha-amylase inhibitors, are intended to be incorporated into the database. Additionally, further member of the *Poaceae* as well as prolamins of maize and rice will be included in the dataset.

## Availability

ProPepper is open access to personal, academic and non-profit use only. The database and analysis platform is available from: https://propepper.net.
